# The power and pain of words: how language matters in responding to patients after harm

**DOI:** 10.3389/frhs.2025.1513670

**Published:** 2025-02-13

**Authors:** E. M. Benjamin, A. Peterson, L. Schweitzer, S. Calcasola, F. Korn, P. Lodato, J. Bradley, C. Hemmelgarn

**Affiliations:** ^1^ Ariadne Labs, Boston, MA, United States; ^2^ Health Policy and Management, Harvard TH Chan School of Public Health, Boston, MA, United States; ^3^ Patient Safety, CommonSpirit Health, Chicago, IL, United States; ^4^ Independent consultant, Reno, NV, United States; ^5^ Clinical Affairs, Hartford Healthcare, Hartford, CT, United States; ^6^ Risk Management, Dartmouth Health, Lebanon, NH, United States; ^7^ Department of Patient Safety and Accreditation, ChristianaCare, Wilmington, DE, United States; ^8^ Department of Medicine and Pediatrics, Geisel School of Medicine at Dartmouth, Hanover, NH, United States; ^9^ Partnership for Patient Safety US, Atlanta, GA, United States

**Keywords:** patient safety, transparency, disclosure & transparency, reconciliation, harm

## Abstract

A change is slowly occurring in the ways healthcare responds to patients after they experience harm. The imperative to be transparent with patients and families has been accepted as a key element of high quality, safe and patient-centered healthcare. The language used to describe the experience of the people impacted by harm events is also evolving, recognizing that certain words can help or hinder the experience of patients affected by harm. The language describing these efforts is shifting from legal and institutional terminology to more inclusive terms recognizing broader groups impacted by harm. We describe the evolution of language regarding harm response and make recommendations for the future of the field. While our observations on language are specific to the terminology used in the United States, the concept of moving to more patient-centered language is universal. Other countries should make similar reviews to use more patient-centered language when discussing patient harm.

## Introduction

A slow but significant change has occurred in how healthcare professionals and organizations respond when something has gone wrong in a patient's care (1). Though our perspective arises from experience in the US healthcare system, inadequate response to patient harm is an international problem that stems from prioritizing institutions over people. One aspect of institutional responses to harm is the language used to navigate the harm response, and in this regard, the timeline of change in the US offers a cautionary but prospectively hopeful tale. We provide a perspective on evolving response structures and their derivative language in the US that may inform thinking on approaches to harm response, and international efforts to refocus on patients and families.

The language describing these efforts and used during conversations with patients and families needs to shift from legal and institutional terminology to more inclusive terms recognizing broader groups impacted by harm. We describe the evolution of the CRP field in the US as a model for others to consider the way language is used in other countries. We share a perspective on the language that is acceptable to patients and make recommendations for the future of the field. While our observations on language are specific to the terminology used in the United States, the concept of moving to more patient-centered language is universal. Other countries should make similar reviews to use more patient-centered language when responding to and discussing patient harm.

## Evolution of communication and resolution programs in the US

Though long honored in theory as an ethical and professional duty (2, 3), policies and structures did not support the practice of disclosure until 2001, when the Joint Commission began to require healthcare facilities to disclose all outcomes of care, including “unanticipated outcomes’, to patients (4). Increasingly the imperative to be transparent with patients and families has been accepted as a key element of high quality, safe and patient-centered healthcare, appearing in national educational frameworks for learners across disciplines (5–8). In 2016, the CANDOR Toolkit was published by the United States Agency for Healthcare Research and Quality (AHRQ), offering organizations a framework to prioritize this approach (9). In 2020, the US National Steering Committee for Patient Safety released 'Safer Together: A National Action Plan to Advance Patient Safety', which recommended that organizations: “implement and maintain programs for providing appropriate ongoing support in the aftermath of harm” (10). The following year, the World Health Organization's “Global Patient Safety Action Plan 2021–2030” emphasized the need to “establish the principle and practice of openness and transparency throughout health care, including through patient safety incident disclosure to patients and families (11).” Most recently the US Centers for Medicare and Medicaid Service (CMS) established a new Patient Safety Structural Measure (PSSM) requiring Medicare participating organizations to attest to the fact they have a robust Communication and Resolution Program (12). New toolkits and other resources have been developed to support transparency, and in the US, hundreds of healthcare organizations have embraced this effort (13, 14).

During this policy evolution, the language of transparency has changed in the US. Initially, these approaches used language familiar to the attorneys who were often called-upon to guide decisions in these situations. “Disclosure programs” emphasized how healthcare organizations should “disclose” what was previously held confidentially within the legal walls of the organization. Soon, transparency was tied to making early offers of financial compensation to patients and families after harm, leading them to be called “Disclosure and Offer” programs (15, 16). Now, the language describing these efforts is shifting from legal and institutional terminology to more inclusive terms that recognize broader groups impacted by harm. The most common current term is “Communication and Resolution Programs (CRPs)” (17, 18) yet patients, their families and healthcare providers do not agree on the best term to describe or to support this activity.

Language describing components of the healthcare industry is constantly evolving. For example, the field of patient safety is a decades-old addition to the millenia-old practice of medicine itself, though its principles have existed in many forms. The relationship between providers and patients is evolving from “doctor knows best,” to a co-production model that instead asks: “how can we work together to achieve a desired state of health for all?” (19). Patient safety advocates have called to move from institutionally-biased terms toward people-centered language when supporting patients and families after harm (20, 21). Some medical educators have heeded this call with new frameworks for practice and iteration (22–25).

While the fundamental elements of a CRP provide a blueprint for healing, how organizations articulate the process is a source of variability and vulnerability. Although each harm context is unique, the nature of communication and the language used matters for all stakeholders (26, 27). For example, one of the definitions for the word *resolution* is “a solution, accommodation, or settling of a problem, controversy, etc” (28). but within the context of a CRP, this raises questions about who is being served by, or experiencing, “resolution.” The patient or family member who suffered a catastrophic, life-changing event from medical harm is unlikely to reach a sense of resolution (20) compared to a member of a risk management or claims department, who might identify resolution as the moment at which compensation has been accepted, and the file is closed.

When harm has occurred, words have a greater power to heal and harm (29). Words are never more crucial to patients and families than after they have suffered a medical error (21, 30, 31). In modern CRPs, there is growing recognition that terms and words created by institutions, rather than the people impacted by the harms of healthcare, miss the perspective of patients and families and may paradoxically alienate them from a process designed to include them. Patients and families will tell you there is rarely a resolution (21). Language has countless nuances, and it is impossible to reach universal consensus on words that resonate equitably with the values of all parties. The goal should be to use words and terms that are patient- and family-focused, avoiding the bygone era of institutional and legal jargon. Words and language should not further hurt injured patients and families, but instead reflect and respect their experience. This will be accomplished when those working in this space collaborate with patients and families to co-create a shared dialect.

In this paper, we review the language of responding to harm often used in the US, and consider language that best aligns with the priorities of patients, families, providers and healthcare organizations. Our goal is to support organizations to use words that align with and advance reconciliation programs in the service of patient care and societal trust. We desire to promote language that supports patients and the system after harm events—specifically not to exacerbate a harm event with insensitive language (29, 30) but to support the process and a collaboration between patients and providers (19, 32, 33).

The authors have all participated in PACT, the Pathway to Accountability, Compassion and Transparency, a US-based learning collaborative sponsored by Ariadne Labs at Harvard School of Public Health, The Collaborative for Accountability and Improvement at the University of Washington, and the Institute for Healthcare Improvement, IHI (14). The PACT collaborative enlists dozens of hospitals annually to renew their commitment and receive implementation coaching regarding their Communication and Resolution Programs (CRPs). In our work to support health systems to implement CRPs, we have worked with providers, patients, patient advisory councils and professionals managing patient safety events. Through these discussions, we have asked these individuals what language they prefer in communication after a harm event.

## Perspectives on language

Patterns have emerged around word preference, demonstrating that different stakeholders have unique perspectives on appropriate terms. In our work implementing CRPs, we have recognized how language is used by different stakeholders and discuss the perspectives and acceptance of words around harm events.

In the US, Communication and Resolution Programs (CRPs) have emerged as the approach to harm response, and are often managed in three phases or components of the process. To better explain the language preferences we discuss words in these three overarching themes reflecting each stage of the event response paradigm (9): (1) *communication* and *apology*; (2) learning (r*eview*, *event understanding)*; and (3) *support* and *reparation*. We discuss words that are commonly used in the US so that the reader may appreciate these words and see parallels in the institutional words that may be used in other settings. Our recommendations for alternative words are also specific to the US, but our goal is that these will stimulate a selection of more patient-centered words that can be used in other settings.

### Communication and apology

Patients experience justifiable fear and real physical and emotional effects after being harmed by the health system (34, 35). They desire to be told what occurred in a transparent and compassionate way (36). We believe that the term “*disclosure”* implies that individuals or an institution has been hiding information, and may continue to do so. If patients and families feel information is being withheld or selectively shared, they may withhold their own concerns and experience, adopt a wary stance, and interpret subsequent discussions through a skeptical lens. This is the opposite of bidirectional communication as a foundation of reconciliation, and may have equity implications if the patient or family has experienced other structural barriers to quality care.

*Harm* to a patient is just that—a patient is hurt by care or treatment intended to help them. Yet we contend that the term “*harm,*” while straightforward and accessible to patients, has a different connotation (and less inclusive criteria) for the risk, claims, provider, and the legal community. This may relate to a combination of historical practices, state laws, or prior experiences with misunderstanding of the term's use to imply culpability and portend malpractice proceedings. For clinicians and patient safety specialists, an *unexpected outcome* describes harm in the course of care, but recognizes the event may fall within the standard of care, representing a known complication. Patients also consider “emotional harm” when they are poorly treated or disrespected by the health system. This broader definition of harm is often lacking in health systems language. These variations of context help to explain discrepancies in language preferences.

Even the word “*apology*” is fraught. The multiple meanings of “sorry” highlights the importance and challenge of getting language right. An apology may be interpreted as a hollow gesture if it is incomplete or insincere (37). The varied meanings of, “I am sorry” or “I apologize” can be illustrated in this statement: “I'm sorry your dog died” (empathy) which is entirely different from: “I apologize for hitting your dog with my car causing him to die” (accountability).

Patients and families desire “*accountability”,* and may want to hear that term used during communication regarding harm events. Patients and families want accountability from their care team even as they realize the system can often be the culpable cause of the harm. Patients want providers to take “responsibility” to acknowledge harm and to improve the care. Providers, on the other hand, may be fearful of the word “accountability” as it implies blame to them. Providers need to move accountability to a broader definition that includes responsibility from a system perspective, not individual blame. In fact, while providers do need to be accountable to patients, they need even more to be responsible for improvement of the system (38).

### Learning

In our collective view, Institutional words such as “*unanticipated event*,” “*root cause analysis*,” and “*investigation*,” may be perceived as obfuscating and promoting a defensive stance.

The word “investigation” can conjure images and thoughts of wrong-doing, police forensics, and cataloging evidence; *crimes* are investigated. However, a simple phrase of “we are concerned about how this happened to you and are committed to learning how we can prevent this from occurring again” implies a desire to understand and fix. All stakeholders clearly understand and accept the term “learning.”

In the absence of context and specifics, the vagueness of an “unanticipated event” may play into a patient's and family's fears about the unknown. Hearing that an “unanticipated event” has occurred may lead patients and their families to wonder “why wasn't it anticipated?” “What event are we talking about?” “Was it a single event or multiple things that happened?”.

We contend that even the term “Root Cause Analysis,” is a highly institutional and technical term that while helpful in the learning process for the organization, can be off putting to patients and families who experience the harm. Root Cause Analysis may make them feel as though they are a number in the system, and not a human who suffered.

### Support and reparation

The term “Resolution” implies the event and its impact have been permanently resolved. However, many patients and families feel the physical or emotional impact of the harm for a lifetime (39). From our experience, patients and families logically prefer the words “reconciliation” or “reparation” over “resolution.” A reconciliation or reparation allows for ongoing dialogue focussed on supporting the patient and family in the aftermath of an event. Reconciliation may involve financial offers, and ongoing emotional support providing ways for patients and families to stay involved in the learning and prevention. These terms imply continuous support and repair of physical or emotional harm while rebuilding relationships.

Patients recognize the impact to a clinician of being involved with a harm event, but are skeptical of the term “*second victim*.” “Second Victim” has been used to describe the complex impacts experienced by clinicians who have contributed to *patient harm,* and is a term used for decades by healthcare organizations to generate awareness and support for involved caregivers (40, 41). This term, more culturally accepted by physicians, is frowned upon by patient advocates who desire to keep the focus primarily on the true victims, the harmed patients (42). The derivative term “third victim”—denoting impact on safety specialists and others in the organization who review harm events as part of their daily work—may be important for professional well-being, yet “third victim” may also appear from the patient/family perspective to diminish the focus on the originally harmed patient, diluting the recognition of life changing harms (43, 44). More organizations are using the term *Care for the Caregiver* (though even this term may not adequately consider inclusion within the team-based care models of contemporary medicine). We believe that patients and families are accepting of this term because they want their caregivers to receive the support and care they deserve, yet they believe there is truly one victim: the patient who was harmed.

In American health care, the terms “settlement” or “offer” may imply “hush” or “payoff” money, rather than financial support for future care. While used in risk management and quality circles, such commonplace impersonal terms may be perceived as callous (45–49). This impact may be felt differently across the continuum of socioeconomic status, health literacy, culture and language.

We contend that when we discuss reparations or reconciliation, using transparent and compassionate language can lessen lingering questions, ongoing anguish and guilt. Discussing compensation/reconciliation is nuanced and we believe that care should be taken to set appropriate expectations about parameters for payment amounts. We have observed that organizations who do it well, avoid terms that suggest closure or resolution, but rather use continued support, compassion and a path to reconciliation.

Patients look to their clinical teams for support and healing. In our opinion, institutions, in turn, need to abandon technical, impersonal terms and demystify the *reconciliation* process for clinical teams, so that those teams can help give patients what they need with confidence. We believe that “*support*” after an event is well understood and desired by patients and the organizations leading CRPs in the US: a step forward for a troubled system.

## Discussion

The field of Communication, Resolution, and Harm Response has grown quickly in the US, and like many areas of health care, is becoming more attuned to a patient-centered approach. With better inclusion has come a more expansive understanding of the harms patients and families experience (34–36, 38, 45–49). Language to properly describe how the health system responds to patient harm is evolving and this global dialogue must adapt to the complexities of culture and language in individual health systems. While there are no “magic words” in the wake of healthcare harm, thoughtful words map tightly to responsive institutional structures to support patients and families.

This approach to use the right words to support patients is related to building patient-centered paradigms reported elsewhere (50). By improving communication after harm, we can reinforce how patients and families can help us to understand how healthcare is safe and where it falls short (51). We believe the right words can engage patients in patient safety and add to the idea of having patients actually support the creation of safe systems (52).

We believe words can be selected to resonate with patients and families and align stakeholders. The goal after harm is to communicate with compassion and minimize risk of offense to the most important party: the patient and family experiencing harm. The language we need to use should be consistent and designed to prevent what has been called “compounded harm:” additional harm to patients and families that occurs after an adverse event has taken place (53, 54). Inappropriate language can compound the harm for all those affected—patients, families, health professionals and organizations—by neglecting to appreciate and respond to the human impacts. We suggest that the risk of compounded harm may be reduced when language responds to the need for healing alongside system learning. For instance, healthcare professionals may be reluctant to speak directly, given underlying fears of lawsuits. This may lead them to use the carefully selected language that is too technical or clinical to be helpful to those harmed by care.

To accomplish this on an international scale requires a perpetual stance of listening and adaptation, taking input from the communities (55), and responding with integrity and openness when we fail. Reliable CRPs with the right language to support patients and learn from events need to avoid adding insult to injury. Insights gathered from many stakeholders we interact with in the US lead us to the English words most fitting this philosophy: communication, harm, learning, accountability, reconciliation, and support (of patients and providers).

Though promising, the US CRPs remain an institutional response structure, and are likely to require ongoing course correction as policy, law and health care delivery pressures evolve, and organizations struggle with equitable implementation. Currently, the threshold for events to enter the CRP process is stringent, and selection criteria are determined by institutions, not by patients. As the patient safety movement expands to consider emotional and dignitary harm in the course of care, the CRP philosophy may have to expand to include events that matter to patients but fall below traditional institutional thresholds of harm.

How can the field move forward? Choosing words is important, but one should recognize that it is often not enough. Patients and clinical teams need something to hold onto beyond theory: a full explanation of the entire CRP process set in practical, local context with consistent terminology is necessary to allay fears of both patients and clinical teams to engage in productive, restorative dialogue. We believe the process needs to go beyond words but also have a highly reliable approach and an infrastructure that is ready to communicate to patients, learn from events and create the proper ongoing support for patients (56).

Finally, every setting will need to determine how to respond with consideration for culture, religion, non-native language will require careful consideration. With the stakes of communication high, proper translation, and cultural humility are paramount.

## Conclusion

Getting our response to harm right is an international ethical imperative. The words we have used to address patient harm are a reflection of structures and incentives that have not always served transparency and healing for our communities. In the US, changes in national health policy and incentive structures are moving into place to support clinicians in responding to harm; bedrock professional ethics have not been enough to overcome structural barriers to talking to patients with integrity. Words are changing to fit these structures; they provide a diagnostic of institutional investment in accountability and transparency. We hope this perspective serves other health systems by turning them inward to look closely at the way they approach patients after harm, both in what they say and what they do to support reconciliation. We should not generate additional harm, either in our dialogues with patients and families, or by privileging words that arose from structures that were not meant to serve patients in the first place. Words and language evolve as awareness, culture, structures, and incentives change. They will continue to evolve, and will require constant vigilance to connect, listen, and compromise, with our ears to improve the support for patients. We need the system to change the terminology such that words align with the language preferred by patients, and the clinical teams that serve them.

## Data Availability

The original contributions presented in the study are included in the article/Supplementary Material, further inquiries can be directed to the corresponding author.

## Glossary

Adverse Event: an injury that was caused by medical management (rather than the underlying disease) and that prolonged the hospitalization, produced a disability at the time of discharge, or both (57).
Emotional Harm: harm to a patient's “dignity” which can be caused by a failure to demonstrate adequate “respect” for the patient as a person [Sokol-Hessner et al. (29)].
Financial Impact: Any reference to prolonged financial issues experienced subsequent to event by patient/family [Ottosen et al. (39)].
Psychological Impact: Any reference to how the event changed the way the patient/family thinks or feels [Ottosen et al. (39)].
Physical Impact: Any reference to prolonged care or physical state experienced subsequent to event by patient/family [Ottosen et al. (39)].
Second Victim: the impact of medical errors on HCPs—especially when there has been an error or the HCP feels responsibility for the outcome [Ozeke et al. (44)].
Social/Behavioral Impact: Any references to how the event changed the way the participant behaved or impacted their social or family life [Ottosen et al. (39)].
